# Effects of 2-Hydroxypropyl-β-Cyclodextrin on the Antioxidant Efficiency of Some Gallic Acid Derivatives in Soybean Oil-in-Water Emulsions

**DOI:** 10.3390/antiox14070887

**Published:** 2025-07-18

**Authors:** Tamara Martínez-Senra, Sonia Losada-Barreiro, Carlos Bravo-Díaz

**Affiliations:** Physical Chemistry Department, Faculty of Chemistry, Universidade de Vigo, 36310 Vigo, Spainsonia@uvigo.es (S.L.-B.)

**Keywords:** cyclodextrins, gallates, inclusion complex, emulsions, lipid oxidation, interfacial concentrations, soybean oil

## Abstract

Cyclodextrins (CDs) have been widely employed as natural host molecules to form inclusion complexes with bioactive molecules such as antioxidants. Their particular spatial configuration, in the form of truncated cones formed through α(1–4) ether linkages of glucopyranose units, makes them very appropriate for the formation of host–guest complexes, modifying their physicochemical properties and their location in multiphasic systems. Here, we investigated the effects of 2-hydroxypropyl-β-cyclodextrin (HPCD) on the efficiency of a series of gallic acid derivatives (propyl (PG), butyl (BG), octyl (OG), and lauryl (LG) gallates) in inhibiting the oxidation of soybean oil-in-water emulsions. For this purpose, we investigated the effects of HPCD on both the kinetics of lipid oxidation and the distribution of antioxidants in the same intact emulsions. The results show that in an aqueous solution, the antioxidants form 1:1 inclusion complexes with HPCD, with inclusion constants ranging from 383 M^−1^ (PG) to 1946 M^−1^ (OG). The results also show that the addition of HPCD to emulsions containing antioxidants does not lead to significant changes in their antioxidant effectiveness, with their efficiency being similar to that when no HPCD molecules are present. The results are interpreted in terms of the blocking effect exerted by the Tween 20 molecules, which act as effective guest competitors capable of removing the antioxidants from the HPCD cavity. The Tween 20 surfactant molecules need to be employed to stabilize the emulsions kinetically. This blocking effect, as a primary consequence, indicates that the interfacial concentration of the antioxidants, which is the region where the inhibition reaction takes place, remains constant; thus, their efficiency is not altered.

## 1. Introduction

**Figure 1:**
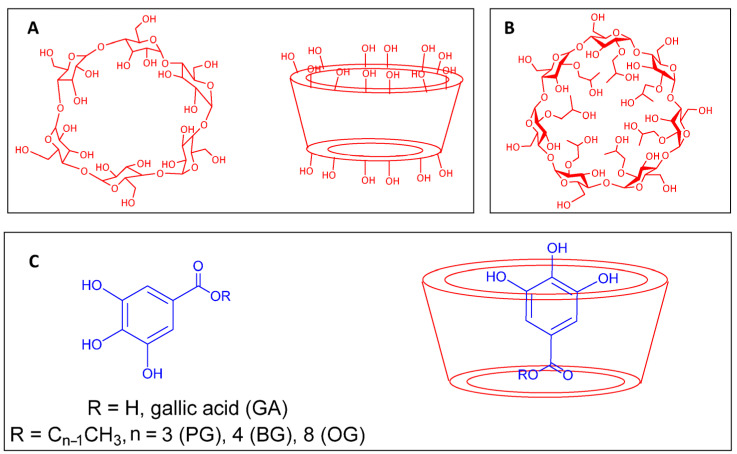
(**A**) The chemical structure of β-cyclodextrin, (**B**) the chemical structure of 2-hydroxypropyl-β-cyclodextrin (HPCD), and (**C**) the chemical structure of gallic acid (its derivatives, used as antioxidants in this study, are shown in the lower left corner of the image). An example of a gallic acid derivative forming an inclusion complex with CDs is depicted on the right side of panel C.

Cyclodextrins are biocompatible natural cyclic oligosaccharides widely used in chemistry, biology, biochemistry, health science, and agriculture [1,2,3,4,5,6,7]. The origin of their many applications is their specific spatial configuration in the form of a truncated cone or “capsule” (Figure 1) with a hydrophobic cavity that encapsulates hydrophobic compounds to form inclusion complexes through non-covalent host–guest interactions. Their derivatives have a wide array of applications in almost all industrial sectors, including foods [5,8].

Natural CDs are composed of six, seven, and eight glucopyranose units (α-CD, β-CD, and γ-CD, respectively), which have identical conformation and are linked to the next by an α-*D*-(1 → 4)-glucosidic bond. Such bonding imparts CDs with three interesting features as shown in Figure 1: (1) all secondary hydroxyl groups are located on one side of the conical cylinder, whereas all primary ones are located on the opposite side; (2) the interior of the cone consists only of a ring of C-H groups, a ring of glucosidic oxygens, and another ring of C-H groups; and (3) the interior of the cavity is less polar than bulk water and has enough space to host hydrophobic molecules [9].

Chemical modifications of naturally occurring CDs are possible and may result in a variety of CD derivatives with enhanced functionally and improved safety profiles such as that of the 2-hydroxypropyl-β-cyclodextrin (HPCD) derivative (Figure 1) employed in this work.

Different investigations reporting that CDs can be employed to control chemical reactivity have advanced their use in catalysis and supramolecular chemistry [10,11]. The preferred position for the guest compound inside the cavity depends on the particular chemical structure and geometry of each guest compound. Sometimes, guest molecules cannot be fully accounted for by a single cyclodextrin molecule, and a second—and even a third—cyclodextrin molecule may bind, leading to the formation of 1:2 and 1:3 inclusion complexes. Moreover, depending on the relative size of the guest and the host molecules, host–guest complexes of 2:1 and 2:2 can be obtained [7].

**Figure 2:**
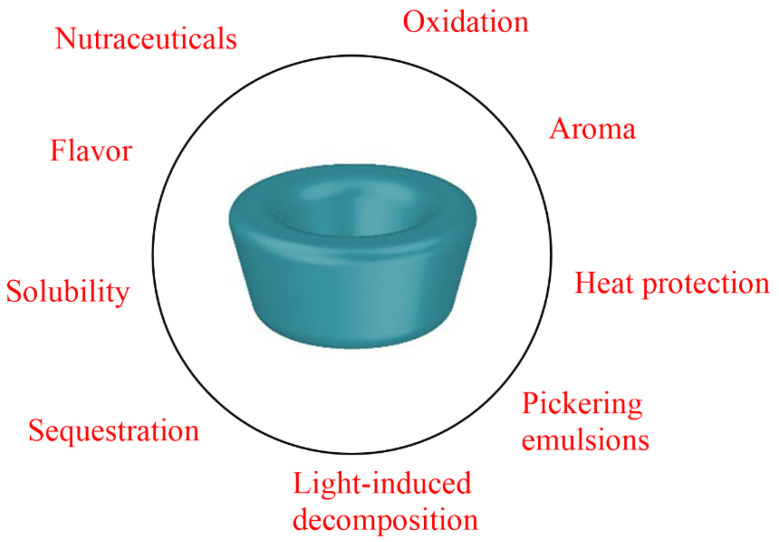
Main applications of cyclodextrins in the food industry.

Cyclodextrins are mainly utilized in the food industry to encapsulate compounds of interest [7,12], with numerous reported applications demonstrating significant enhancements in organoleptic properties (for example, eliminating odors and/or flavors), in the shelf-life of lipid-based products (e.g., Pickering emulsions), and even in the prevention of lipid oxidation (e.g., acting as secondary antioxidants) (Figure 2). Several studies have reported that CDs can modulate the color of foods by increasing the solubility and chemical stability of natural and synthetic coloring products, provoking the inhibition of browning-inducing polyphenol oxidase reactions [10]. CDs have also been reported to form complexes with ingredients that are sensitive to oxygen or oxidizing molecules, preventing these oxidizable molecules (e.g., unsaturated lipids) from undergoing chemical oxidation [13].

In recent last years, the food industry has tried to provide consumers with fortified foods, with this being one of the major research areas that employs antioxidants (AOs) of a lipophilic nature [14]. In spite of the fact that hydrophobic antioxidants such as gallic acid derivatives (propyl (E310) and octyl (E311)) and some flavonoids (e.g., catechin, quercetin) show numerous advantages, water solubility problems limit their use. These solubility problems may be solved, at least partially, by encapsulating them with cyclodextrins. Zarzycki et al. [15] reported on the encapsulation of flavonoids and phenolic acids and their applications in food products and supplements. López-Nicolas et al. [9] presented a comprehensive review of the inclusion complexes formed between antioxidants and cyclodextrins, where the authors highlighted the contradictory data reported in the literature to date on the antioxidant activity of host–guest molecular complexes.

Some studies indicate that cyclodextrins may act as secondary antioxidants, acting synergistically with ascorbic acid and prolonging the life of foods [16]. Li et al. reported on the preparation of propyl gallate–cyclodextrin complexes with improved aqueous solubility and radical scavenging activity, suggesting that the use of cyclodextrins can be an efficient strategy for protecting polyunsaturated fatty acid from oxidation in the food industry [17]. On the contrary, other reports indicate that the formation of antioxidant–cyclodextrin complexes did not lead to a significant increase in antioxidant efficiency in spite of the fact that they increased the aqueous solubility of the investigated hydrophobic antioxidants [18,19].

The question of whether cyclodextrin (CD) inclusion universally enhances antioxidant activity is actively debated, with reports demonstrating that the effects are highly compound-specific and influenced by multiple factors [11,18,20]. The reaction environment (solvent type, polarity) significantly affects the thermodynamics and structure of the inclusion complex, thereby influencing antioxidant efficiency. For example, the Gibbs energy for the complexation and the binding interactions can change with different solvents, altering the efficacy of the AO–CD complex [21].

In previous works, we investigated the formation of inclusion complexes between a number of gallic acid derivatives and cyclodextrins in an aqueous solution, concluding that the antioxidants form 1:1 (AO–CD) inclusion complexes [6,22]. Here, we report on the effects of combining soybean oil-in-water emulsions and cyclodextrins (CDs) on the formation of the inclusion complexes and explore the use of cyclodextrins in a model food-grade emulsion to investigate their effects in preventing lipid peroxidation in both the presence and the absence of a series of gallic acid derivatives antioxidants.

Lipid peroxidation is a major food reaction (Equations (1)–(7)) that takes place everywhere molecular oxygen and unsaturated lipids (LH) are present [23,24]. The higher the number of unsaturations in the chemical structure of lipids, the faster the reaction is. The relative oxidation rates for oleic–linoleic–linolenic acids are approximately 1:70:100 based on oxygen uptake [25,26,27]. This means that polyunsaturated fatty acids (PUFAs), particularly omega-3 (such as linolenic acid), oxidize far more rapidly than monounsaturated fatty acids (MUFAs, such as oleic acid), and much more rapidly than saturated fatty acids such as stearic acid, whose oxidation rate is almost negligible because of spin restrictions.

Initiation

(1) $$\mathrm{In}\overset{k_{\mathrm{ini}}}{\to }{\mathrm{In}}^{•}\;$$

(2) $${\mathrm{In}}^{•}+\mathrm{LH}\to L^{•}+\mathrm{In}\text{-}H\;$$

Propagation

(3) $$L^{•}{+O}_{2}\to {\mathrm{LOO}}^{•}$$

(4) $${\mathrm{LOO}}^{•}+\mathrm{LH}\overset{k_{p}}{\to }{\mathrm{LOOH}+L}^{•}$$

Termination

(5) $${2\mathrm{LOO}}^{•}\overset{k_{t}}{\to }\mathrm{non}\text{-}\mathrm{radical}\;\mathrm{products}\;$$

Inhibition

(6) $${\mathrm{LOO}}^{•}+\mathrm{ArO}\text{-}H\overset{k_{\mathrm{inh}}}{\to }{\;\mathrm{LOOH}+\mathrm{ArO}}^{•}\;$$

(7) $${\mathrm{LOO}}^{•}{+\mathrm{ArO}}^{•}\to \mathrm{non}\text{-}\mathrm{radical}\;\mathrm{products}\;$$

The lipid peroxidation reaction is initiated by means of heat or light or any other radical initiator (In) that may be present in the system (e.g., metal ions) and leads to the formation of a lipid radical L^•^, which immediately reacts with oxygen to give a peroxyl radical LOO^•^ (Equations (1)–(3)). These peroxyl radicals continue the reaction, producing a variety of compounds (LOOH fatty acid hydroperoxides) that lead to the formation of off-flavors, rancidity, and the loss of nutritional value until the reaction is terminated because all the starting material has been consumed or because the reactions of the radicals led to the formation of non-radical products or other radicals that are much less reactive [28]. The peroxidation reaction is usually minimized upon the addition of antioxidants (ArOH) that react with the peroxyl radicals, (Equations (6) and (7)), restoring the lipid and generating a significantly less reactive antioxidant radical, ArO^•^ [28].

Here, we have investigated how combining emulsions loaded with antioxidants and cyclodextrins influences their antioxidant efficiency and modifies their relative concentrations in the interfacial region of the emulsions, which is the reaction site where the inhibition reaction takes place.

We have employed some alkyl esters derived from gallic acid because they are potent antioxidants that play a significant role in inhibiting lipid oxidation by interacting with lipid peroxyl radicals. This process produces a gallate-derived radical, which is much less reactive than the peroxyl radical and does not propagate further lipid damage, extending their shelf-life, and therefore demonstrating their effectiveness in inhibiting lipid peroxidation. The antioxidant activity of gallates is influenced by their molecular structure, particularly the length of the alkyl chain, which modifies their hydrophobicity, leading to substantial changes in their distribution in emulsified systems [29,30]. Medium- and long-chain alkyl gallates (e.g., propyl, butyl, and octyl gallates) exhibit higher antioxidant activity than gallic acid and shorter-chain esters, particularly in emulsified lipid systems [29,30].

## 2. Materials and Methods

### 2.1. Instrumentation

A VWR LA214i analytical balance (precision: ±0.0001 g, VWR lnternational Eurolab, S.L.U.; Llinars del Vallés, Barcelona, Spain) was employed to weigh the samples. pH measurements were obtained with the aid of a Metrohm 713 pH-meter (Metrohm, Madrid, Spain) equipped with a glass electrode and a temperature sensor. The pH-meter was previously calibrated by employing commercial buffer solutions of pH = 4 and pH = 7.

Absorbance measurements were obtained by employing an Agilent 8453 UV-Vis (Agilent, Santa Clara, CA, USA) diode array spectrophotometer equipped with a thermostated multicell carrier. Thermostatization was achieved by employing water from a Julabo F34 (Julabo Gmbh, Seelbach, Germany) thermostat/cryostat, which warrants a precision in T of ±0.1 °C. Quartz absorbance cells with an optical length of l = 1 cm and a volume of 3.5 mL were employed.

Steady-state fluorescence measurements were carried out on a Cary Eclipse spectrofluorimeter (Agilent, Santa Clara, CA, USA) equipped with a Peltier temperature controller by employing 1 cm quartz cell. The excitation wavelength was fixed at 276 nm (excitation slit 20 nm), and the emission spectrum was obtained between 300 and 450 nm (emission slit width 5 nm).

### 2.2. Materials

All chemicals were used without any further purification. Propyl gallate (PG, 99%), butyl gallate (BG, 99%), octyl gallate (OG, 99%), lauryl gallate (LG, 99%), 2,2-diphenyl-1-pycrilhydrazyl (DPPH^∙^), and the coupling agent N-(1-naphthyl)ethylenediamine (NED) were purchased from Sigma-Aldrich. 2-Hydroxypropyl-β-cyclodextrin (HP-CD) was purchased from Cyclolab LTD (CycloLab Cyclodextrin Research and Development Laboratory Ltd, Budapest, Hungary). Millipore (Merck Life Science S.L.U., Madrid, Spain) Milli-Q grade water (conductivity < 0.1 mS·cm^−1^) was used to prepare the aqueous solutions. A 0.04 M citric acid–sodium citrate buffer solution (pH 3.0), prepared with reagents from Acros Organics (Geel, Belgium), served as the buffer. Soybean oil, kindly provided by Aceites Abril (Ourense, Spain), was stripped from its endogenous antioxidants by passing the oil through a chromatographic glass column packed with alumina previously activated in an oven at T = 200 °C for 24 h. Stripped soybean oil composition (%*w*/*v*) was 13% saturated fatty acids, 28% oleic acid (18:1), 54% linoleic acid (18:2), and 5% linolenic acid (18:3).

4-Hexadecylbenzenediazonium tetrafluoroborate (16-ArN_2_BF_4_) was synthetized under non-aqueous conditions from commercial 4-hexadecylaniline (Aldrich (Darmstadt, Germany), 97%) [31].

### 2.3. Emulsion Preparation

The 1:9 oil-in-water emulsions were prepared by mixing 1 mL of stripped soybean oil and 9 mL of buffered aqueous solution (citric–citrate buffer 0.04 M pH 3.0) and different amounts of the surfactant Tween 20 (Φ_I_ = V_surfactant_/V_emulsion_ = 0.005 − 0.04). Emulsions prepared without antioxidants were used as controls. In the others, the antioxidants GA, PG, OG, and LG were added to the aqueous (GA, PG) or oil (OG, LG) phases. The mixtures were stirred for at least 60 s at 20000 rpm at room temperature by employing a high-speed rotor (Polytron PT 1600 E, Kinematica, Fisher Scientific S.L., Madrid, Spain). All emulsions were prepared in triplicate and reported values are average.

### 2.4. Methods

#### 2.4.1. Determining the Inclusion Constant (*K*_c_) Values of Antioxidants with Cyclodextrins

It is well documented that the hydrophobic cavities of cyclodextrins are capable of hosting molecules with some hydrophobic degree in their interior. The stoichiometries of such complexes are, most frequently, 1:1 AO–CD, but complexes with higher stoichiometries (1:2, 2:1, and even 1:3) have been described. The formation of these complexes with HPCD is described by Equation (8) where *K*_c_ is the inclusion equilibrium constant.

(8) $$nAO+mCD⇌AO_{n}-CD_{m}$$

**Figure 3:**
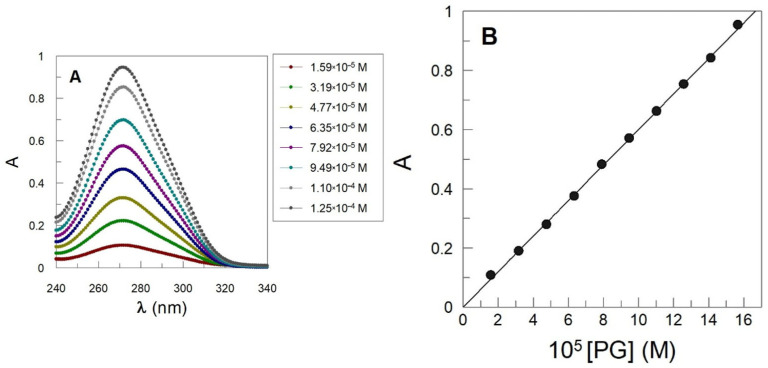
(**A**) Absorption spectrum of aqueous PG buffered solutions (citric–citrate 0.04 M pH = 3.00) and (**B**) Beer–Lambert plot of the variation in the absorbance (λ = 270) with [PG].

Determination of the inclusion constants is usually carried out through the analysis of the changes in physical parameters of the “free” and “included” guests as a consequence of the differential solvation properties of the aqueous solution and the interior of the cavity. For the most hydrophilic antioxidants (GA, PG), we exploited the differential solubility of the host molecules according to the Higuchi and Connors phase solubility method [32]. Briefly, an excess of the antioxidant is added to vials containing increasing amounts of cyclodextrin. The vials are allowed to reach equilibrium, and the total antioxidant concentration is determined by employing a suitable analytical technique—in our case, UV-vis spectroscopy (Figure 3). Details are given elsewhere [22].

This method could not be employed with OG because OG is not water soluble. In this case, we took advantage of its photochemical properties and employed a steady-state fluorescence method. Briefly, an aliquot (100 μL) of a methanolic solution of octyl gallate (0.007 M) was introduced in different 25 mL volumetric flaks containing increasing concentrations of HP-CD (0–0.015 M) and prepared in buffered solution (citric–citrate 0.04 M, pH = 3.5) in the absence and in the presence of Tween 20 (0.0039 M). The total stoichiometric concentration of OG was 7·10^−5^ M after dilution. The mixtures were located in an orbital shaker (T = 25 °C (±1 °C), 470 rpm) in the dark for at least 1 h to allow samples to reach thermal equilibrium.

The inclusion constants were determined by using the Benesi–Hildebrand [33] Equation (9), where I_f0_ and I_f_ stand for the fluorescence intensities of OG in the absence and in the presence of HPCD, respectively, I_f∞_ is the fluorescence intensity value of OG for the highest concentration of HP-CD, *K*_C_ is the inclusion constant for the formation of the complex OG-CD, and n represents the stoichiometry of the formed complex.

(9) $$\frac{1}{I_{f}-I_{f_{0}}}=\frac{1}{I_{f_{\infty }}-I_{f_{0}}}+\frac{1}{\left(I_{f_{\infty }}-I_{f_{0}}\right)K_{C}{\left[CD\right]}^{n}}$$

Equation (9) predicts that the double-reciprocal plot of 1/(I_f_ – I_f0_) vs. 1/[CD]^n^ should be linear, and the inclusion constant *K*_C_ value can be obtained from the ratio between the intercept and the slope of the straight line.

#### 2.4.2. Distribution of Antioxidants: Determination of the Partition Constants P_W_^I^ and P_O_^I^

**Figure 4:**
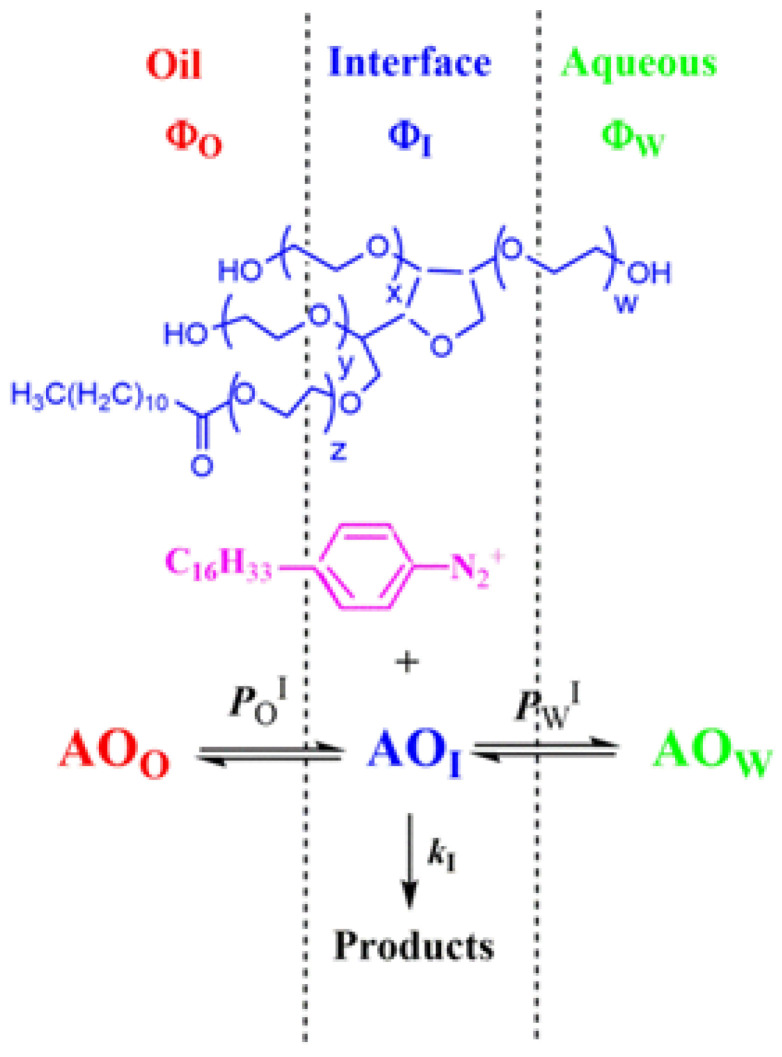
Illustrative section of an emulsion droplet showing oil, interfacial, and aqueous regions. Surfactant Tween 20 stabilizes the emulsion kinetically. Antioxidants are distributed thermodynamically according to their solubilities between the oil, interfacial, and aqueous regions. Their distribution is described by the partition constants *P*_W_^I^ and *P*_O_^I^. Figure also shows the location of the reactive moiety of the 16-ArN_2_^+^ chemical probe used to determine *P*_W_^I^ and *P*_O_^I^ values (see text).

Emulsions contain three regions (oil, interface, and aqueous) with distinct solvent properties, and antioxidants are partitioned between them to different extents depending on their hydrophobicity (Figure 4). To prevent disturbance of the existing equilibria, the partition constants were evaluated in the intact emulsions using a well-established chemical method based on the reaction between a chemical probe, 4-hexadecylbenzenediazonium (16-ArN_2_^+^), and the antioxidants. Kinetic results are then analyzed using a pseudophase kinetic model.

The method, along with its assumptions, advantages, and limitations, are described in detail elsewhere [28,31]; only the relevant parts will be summarized here. 16-ArN_2_^+^ is specifically designed as a water-insoluble chemical probe due to its long hydrophobic tail but with an ionic head group that makes it oil-insoluble. Thus, the reactive (-N_2_^+^) group is located exclusively in the interfacial region. The rate of its reaction with the antioxidant depends on the effective concentration of the antioxidant in that region, which, in turn, depends on its distribution (Figure 4). The partition constants of the antioxidant between the water interfacial (*P*_W_^I^) and the oil interfacial (*P*_O_^I^) regions are described by Equations (10) and (11), respectively, where the magnitudes within parenthesis indicate the local concentrations given in moles per liter for a specific region.

(10) $$P_{W}^{I}=\frac{{(\mathrm{AO}}_{I})}{{(\mathrm{AO}}_{W})}$$

(11) $$P_{O}^{I}=\frac{{(\mathrm{AO}}_{I})}{{(\mathrm{AO}}_{O})}$$

By employing the formalism of the pseudophase kinetic model, mathematical equations describing the relationships between the measured rate constant (*k*_obs_) for the reaction involving 16-ArN_2_^+^ and the AOs and the partition constants can be derived, as was shown in our previous published work [31,34]. Equation (12) was employed for the AOs, which are distributed between the three regions. Equation (12) can be simplified to Equation (13) for those antioxidants that only partition between the oil and the interfacial regions.

(12) $$k_{\mathrm{obs}}=\frac{\left[{\mathrm{AO}}_{T}\right]k_{I}P_{W}^{I}P_{O}^{I}}{\Phi_{O}P_{W}^{I}{+\Phi }_{I}P_{W}^{I}P_{O}^{I}{+\Phi }_{W}P_{O}^{I}}$$

(13) $$k_{\mathrm{obs}}=\frac{k_{I}\left[{\mathrm{AO}}_{T}\right]P_{O}^{I}}{\Phi_{I}P_{O}^{I}+\Phi_{O}}$$

With the partition constants known, the percentage of AOs at the interface can be determined, and the effective concentration of AOs in each region can be determined straightforwardly by employing Equations (14)–(16) [28,34].

(14) $${\%\mathrm{AO}}_{I}=\frac{{100\Phi }_{I}P_{W}^{I}P_{O}^{I}}{\Phi_{O}P_{W}^{I}{+\Phi }_{I}P_{W}^{I}P_{O}^{I}{+\Phi }_{W}P_{O}^{I}}$$

(15) $${\%\mathrm{AO}}_{I}=\frac{{100\Phi }_{I}P_{O}^{I}}{\Phi_{I}P_{O}^{I}{+\Phi }_{O}}$$

(16) $$({\mathrm{AO}}_{I})=\frac{\left({\%\mathrm{AO}}_{I}\right)\left[{\mathrm{AO}}_{T}\right]}{\Phi_{I}}$$

#### 2.4.3. Determining the Observed Rate Constants (*k*_obs_) for the Reaction Between Chemical Probe and Antioxidants in Soybean Oil-in-Water Emulsions

**Figure 5:**
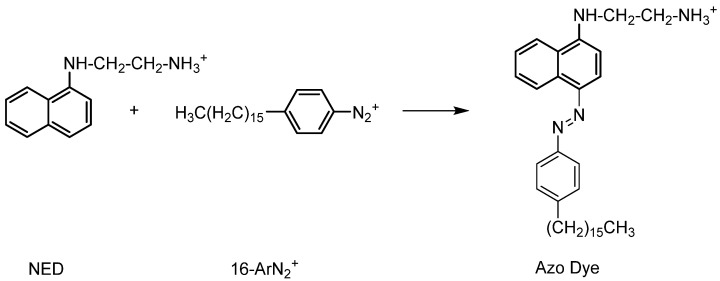
Reaction between the chemical probe (16ArN_2_^+^) and the coupling agent (NED) to give a stable azo dye.

The application of the pseudophase kinetic model described above requires the determination of the variation in the observed rate constant (*k*_obs_) for the reaction between the chemical probe (16ArN_2_^+^) and the antioxidants with the surfactant concentration. Because emulsions are opaque, a special protocol based on a derivatization method has been used to determine the *k*_obs_ values [35]. Briefly, unreacted arenediazonium ions were rapidly quenched by employing a suitable coupling agent (N-(1-naphthyl)ethylenediammine, NED), leading to the formation of a stable azo dye (Figure 5). The absorbance of the azo dye formed is proportional to the concentration of the unreacted 16ArN_2_^+^ present in the reaction mixture. Further information regarding the experimental procedure is provided in a previous publication [35].

**Figure 6:**
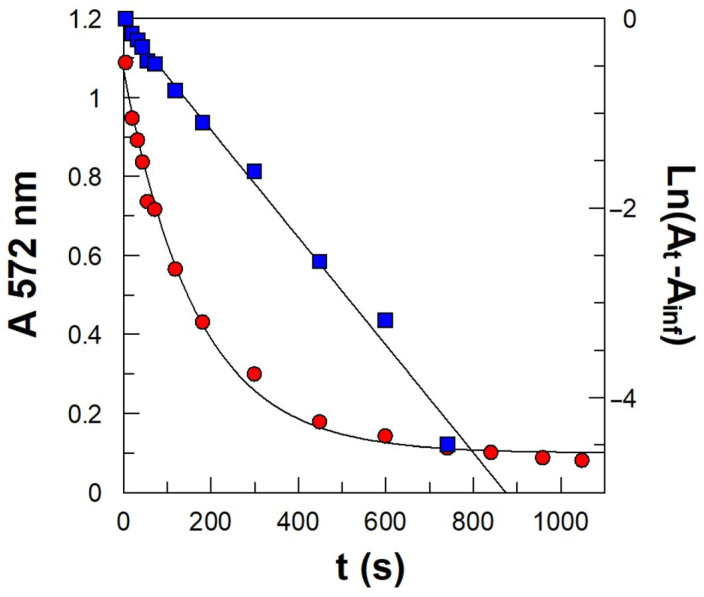
Variation in the absorbance values of the azo dye obtained from the reaction between unreacted 16ArN_2_^+^ and the coupling agent NED with time in the presence of propyl gallate (red circles). The solid lines represent the fitting of experimental data to the integrated first-order equation (blue squares). Experimental conditions: 1:9 soybean o/w emulsion stabilized with Tween 20 (Φ_I_ = 0.015). The aqueous phase was buffered (citric–citrate buffer 0.04 M pH 3.0). T = 25.0 °C; [16ArN_2_^+^] = 2·10^−4^ M; [NED] = 0.02 M; [PG] = 3·10^−3^ M.

A typical kinetic plot is shown in Figure 6. The *k*_obs_ values were determined by fitting the absorbance–time data pairs to the pseudo-first-order kinetic equation (Equation (17), where A_t_, A_∞_, and A_0_ stand for the absorbance values at time t, infinitive time, and zero time, respectively).

(17) $$\ln\left(A_{t}-A_{\infty }\right)=\ln\left(A_{0}-A_{\infty }\right)-k_{\mathrm{obs}}t$$

#### 2.4.4. Kinetics of Lipid Oxidation

The kinetics of the lipid peroxidation of soybean oil-in-water emulsions were monitored by determining the variation in the formation of conjugated dienes (CDs) with time according to the AOCS Official Method Ti 1a 64 in both the absence (control) and the presence of antioxidants (stoichiometric [AO] = 10^−4^ M). Briefly, 1:9 O/W emulsions (Φ_I_ = 0.008) were transferred to 25 mL screw-capped erlenmeyer flasks and were placed in an orbital shaker (Incubator Heidolph 1000 orbital stirrer, Heidolph Scientific Products GmbH, Schwabach, Germany) equipped with a Heidolph thermostat 1010 to the control temperature at 148 rpm and T = 60 °C (±1 °C) in the dark.

Aliquots (25 µL) of the emulsions were removed periodically and diluted to 10 mL with 2-propanol, and the absorbance was measured at λ = 233 nm. The percentage of conjugated dienes (%CD) was determined, as described in the literature, by employing Equation (18), where C_oil_ stands for the concentration of soybean oil in the total volume of the emulsion (density for soybean oil = 0.93 g/mL; T = 25 °C).

(18) $$\%CD=1.0769\frac{A_{233nm}}{C_{oil}(g/L)}$$

#### 2.4.5. Determination of the Radical Scavenging Activity

The radical scavenging activity of gallates was obtained by employing the free radical 2,2-diphenyl-1-pycrilhydrazyl DPPH^•^ and by following the method described by García-Perez et al. [6]. The absorbance decrease in DPPH^•^ (λ= 515 nm) was monitored in the absence and the presence of CD for different concentrations of AOs ([AO] = 5·10^−3^ − 1.3·10^−2^ M) in an aqueous–methanolic solution (5:5, *v*/*v*) at T = 25 °C. Auxiliary experiments showed that the extinction molar coefficient of DPPH^•^ was equal in the absence and in the presence of CDs, suggesting that DPPH^•^ molecules were not included inside the cavity of the CD. The radical scavenging activity was measured in terms of the EC_50_ values, defined as the gallate concentration required to decrease DPPH^•^ radicals by half.

## 3. Results and Discussion

### 3.1. Radical Scavenging Activity of Gallates in Aqueous/Methanolic Solution

Before assessing the efficiency of the antioxidants (AOs) in soybean oil-based emulsions, we first needed to confirm whatever the presence of HPCD had an impact on their reactivity. To address this, a previous study examined the antiradical activity of these compounds in bulk solution using the DPPH^•^ assay in the absence and the presence of HPCD.

The results in Table 1 reveal two important findings: (1) the reactivity of the antioxidants with the DPPH radical differs slightly according to the length of the alkyl chain. This indicates that the reactive polyphenolic structure, rather than the length of the alkyl substituent, primarily determines its ability to scavenge DPPH radicals; (2) in the absence of cyclodextrins, the average EC_50_ value for the gallates (propyl, butyl, and octyl gallate) was 7.37 ± 0.3 μM. When 1.1·10^−2^ M HPCD was present, the differences in the EC_50_ values were less than 30% when increasing alkyl chain length, averaging 6.75 ± 0.1 μM. Since all gallates form inclusion complexes with HPCD, and since the EC_50_ values are maintained upon its addition, it can be concluded that the radical scavenging activity of the complexed antioxidants is similar to that of the uncomplexed compounds in aqueous–methanolic solution (5:5, *v*/*v*) at T = 25 °C.

### 3.2. Antioxidant Efficiency in Soybean Oil-in-Water Emulsions

**Figure 7:**
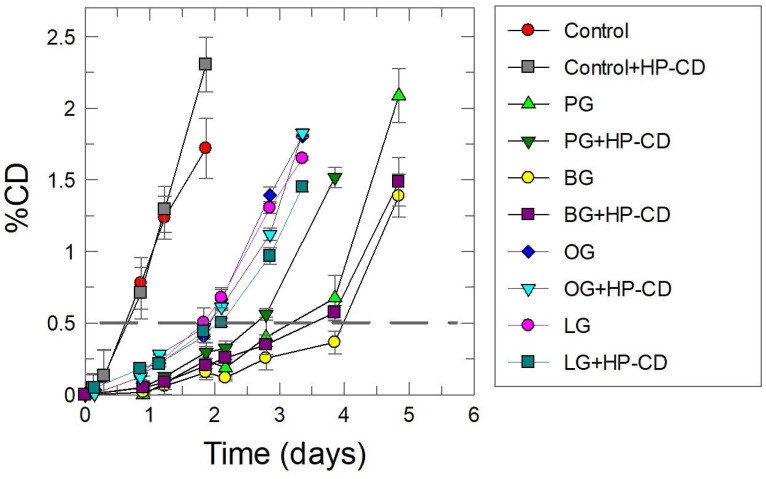
Kinetic profile of the variation in the formation of conjugated dienes (%) in 1:9 (O/W) soybean oil-based emulsions in the absence and the presence of HP-CD and different AOs (aqueous phase: citric–citrate buffer 0.04 M; pH = 3.0; Φ_I_ (Tween 20) = 0.008; [HP-CD_T_] = 0.013 M; [PG_T_] = [BG_T_] = [OG_T_] = [LG_T_] = 0.1 mM; 148 rpm; T = 60 °C).

The antioxidant efficiency of gallates in the absence and in the presence of HP-CD was monitored by determining the formation of conjugated dienes (CDs) with time according to the AOCS Official Method Ti 1a 64. Figure 7 shows the lipid oxidation kinetics profiles in the absence (control) and the presence of AOs under different experimental conditions. In the presence of antioxidants, CDs are formed slowly (compared to their formation in the absence of antioxidants) and are followed by a rapid peroxidation step. Such kinetic profiles are commonly interpreted in terms of the inhibition reaction (lag phase corresponding to the “slow” formation of primary oxidation products, Equation (3)) that proceeds up to the consumption of most of the antioxidant. The subsequent faster step is a consequence of the lack of antioxidants and proceeds at a rate equal to that in the absence of antioxidants (control experiment).

**Figure 8:**
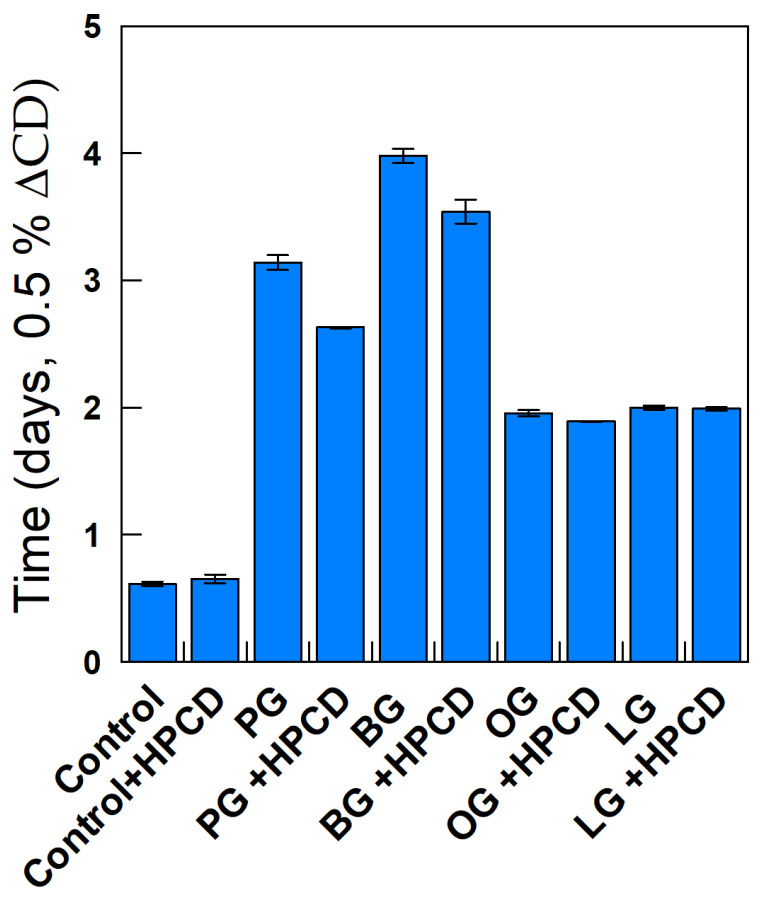
Time needed to reach an increase of 0.5% in the production of CDs. Data are extracted from Figure 7.

The relative efficiency of the antioxidants under the different experimental conditions was quantified by measuring the time needed to increase the CD content by 0.5% (dashed line in Figure 7). We have previously shown that these values are equal to those for the induction period determined from the intersection point of the two linear segments defining the inhibited and uninhibited (propagation) steps [28]. The results obtained under different experimental conditions are displayed in Figure 8.

In the absence of antioxidants, the oxidation of lipids in 1:9 soybean emulsions (Equations (1)–(4)) is very fast, and an increase of 0.5% in the production of CDs is achieved in less than 10 h. The addition of HPCD has no effect on the kinetics of oxidation, indicating that the presence of cyclodextrins do not show either pro-oxidant or inhibition effects on the oxidation reaction.

Upon the addition of antioxidants, Equations (6) and (7), take place, becoming competitive with reaction showed in Equation (4), and as a consequence, the oxidative stability of the emulsions increases. The extent of the inhibition of the peroxidation reaction depends on the nature of the AO, with the relative efficiency order being LG ≈ OG < PG < < BG. This variation in efficiency with the hydrophobicity of the antioxidant (the so-called “cut-off” effect) has previously been observed by us and others and has been interpreted in terms of the variation in the effective concentration of the antioxidant in the interfacial region of the emulsions [28].

The addition of HPCD to emulsions containing PG and/or BG decreases the oxidative stability slightly (when compared to that in its absence) but has a negligible effect when emulsions are loaded with OG or LG. Overall, the results show that the addition of CDs to emulsions loaded with antioxidants has a negligible effect on their oxidative stability, suggesting that the presence of CDs has little effect, if any, on the effective concentration of the antioxidants in the interfacial region of the emulsions.

### 3.3. Distribution of Antioxidants in Intact Soybean Oil-in-Water Emulsions: Effects of Cyclodextrins

**Figure 9:**
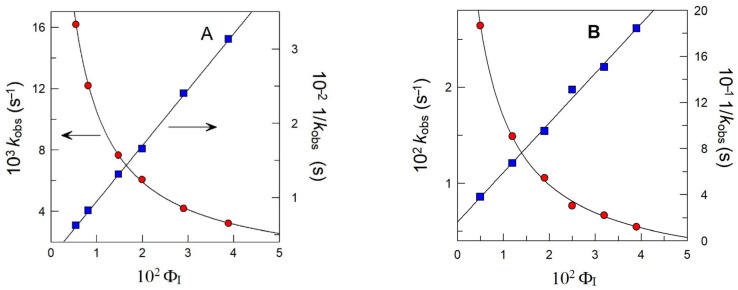
Variation in *k*_obs_ (red circles) and 1/*k*_obs_ (blue squares) values and their fitting to Equations (12) and (13) and their reciprocals: (**A**) PG (in the absence of HP-CD); (**B**) OG (in the absence of HP-CD)). Experimental conditions: 1:9 (O/W) soybean oil/Tween 20/citric–citrate buffer 0.04 M pH = 3.0; [16-ArN_2_^+^] = 2 × 10^−4^ M; [NED] = 0.02 M; [PG_T_] = 3 ×10^−3^ M; [OG_T_] = 2 × 10^−3^ M; T = 25 °C.

The distribution of the antioxidants in the intact emulsions was assessed through the analysis of the variation in the observed rate constants (*k*_obs_) for the reaction between the chemical probe 16-ArN_2_^+^ and the AOs with the emulsifier volume fraction Φ_I_ (Figure 9). Details of this method can be found elsewhere [28,31]. For all AOs, *k*_obs_ values decrease asymptotically 3–6 times upon increasing Φ_I_ from Φ_I_ = 0.005 to Φ_I_ = 0.039. The experimental (1/*k*_obs,_ Φ_I_) pairs of data were fitted to Equations (12) (PG and BG) and (13) (OG and LG). The partition constants between the oil interfacial (*P*_O_^I^) and aqueous interfacial (*P*_W_^I^) regions were determined, and their values are displayed in Table 2.

*P*_W_^I^ values for PG are significantly lower than those estimated for BG, as would be expected from their differential solubilities in water [29,30]. The *P*_W_^I^ values for OG and LG could not be determined as they are water insoluble. The *P*_O_^I^ values decrease upon increasing the hydrophobicity of the antioxidant in accordance with the expected improvement in their solubility in oil.

**Figure 10:**
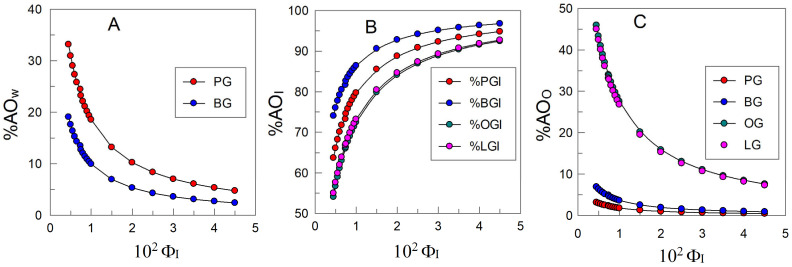
Variation in the percentage of AOs in the aqueous (**A**), interfacial (**B**), and oil (**C**) regions of soybean emulsions in the absence of HPCD.

The percentages of the AOs in the aqueous, interfacial, and oil regions were obtained by employing the partition constant values in Table 2. The percentage of AOs in the interfacial regions was determined by employing Equation (14) (PG and BG) and Equation (15) (OG and LG). Similar equations were employed to determine the percentages in the aqueous and oil regions. Figure 10 shows their distribution as a function of the surfactant volume fraction.

Figure 10 shows that OG and LG are only distributed between the oil and interfacial regions; meanwhile, PG and BG are present in different proportions in the three regions. The percentage of AOs in the aqueous and oil regions decrease upon increasing Φ_I_, contrary to that in the interfacial region, which increases from > 50% (Φ_I_ = 0.005) to > 90% when Φ_I_ = 0.045. At any particular Φ_I_ value, there is no correlation between the percentage of the AO in the interfacial region and its hydrophobicity, with the order being %BG_I_ > %PG_I_ > %LG_I_ ≈ %OG_I_, in keeping with previous results. Similarly, the results revealed a negligible effect of the presence of HPCD on AO distribution between the different regions of the emulsion.

### 3.4. Effective Concentrations of Antioxidants in the Interfacial Region of Emulsions and Concentration-Efficiency Relationships

**Figure 11:**
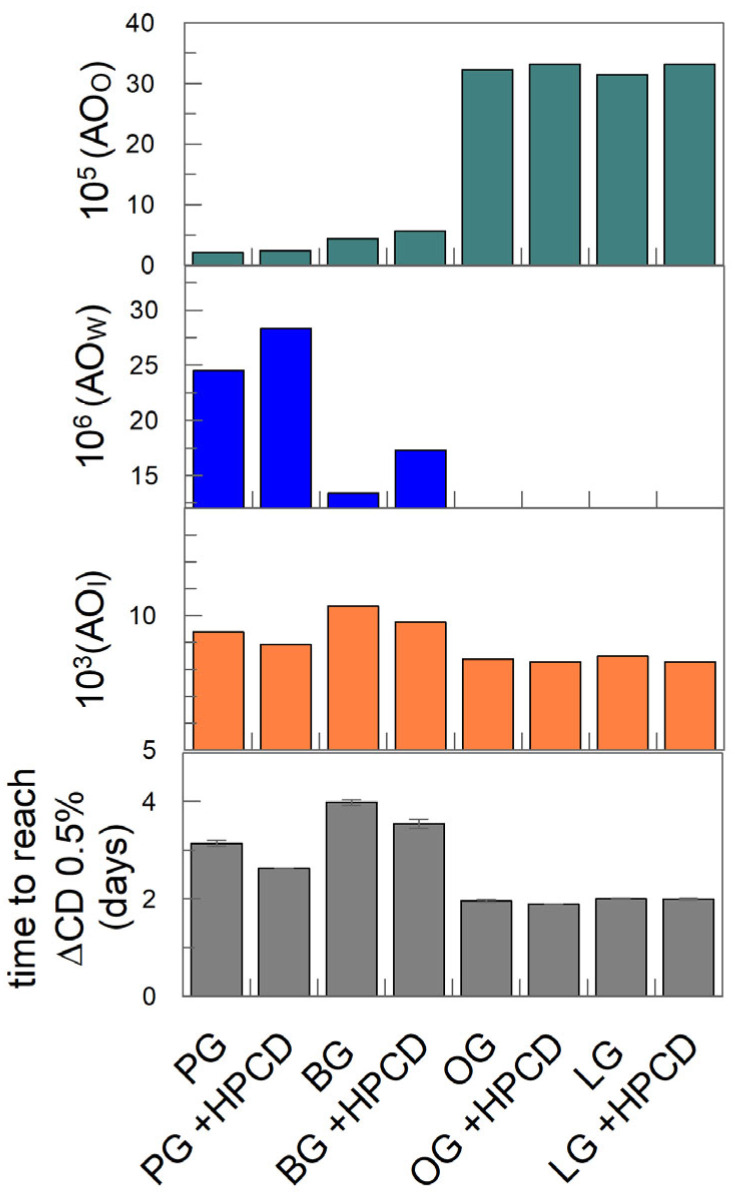
Effective concentration (M) of gallates in the different regions (aqueous, interfacial, and oil) of 1:9 O/W soybean oil-in-water emulsions in the presence and absence of HPCD and the correlation with the determined antioxidant efficiency in the same intact emulsions. Effective concentrations are expressed in terms of moles per liter of the particular region. Φ_I_ = 0.008; [AO_T_] = 1 × 10^−4^ M.

Figure 11 shows the effective concentrations for gallates (AO) in the three regions of the emulsified system in the absence of HPCD. No significant difference in these values was detected either in the presence or absence of HPCD. The effective concentrations in each region are calculated, taking into consideration the number of moles and the volume of the particular region. This effective concentration is different from the stoichiometric concentration [AO]—i.e., the concentration with respect to the total emulsion volume, which was [AO] = 10^−4^ M. The interfacial volume represents only a small portion of the total volume of the emulsion, and the percentage of antioxidants in that region is much higher than that in the others (Figure 10). Therefore, the local concentrations of the AOs at the interface are much higher (~80–110 fold) than their stoichiometric counterparts; meanwhile, the effective concentrations in the aqueous and oil regions are ~3–7 and ~3–5 times lower than the stoichiometric concentration, respectively. This fact is due to the fractions of the antioxidants in those regions being much lower than in the interfacial region and their volumes being much larger than in the interfacial region.

The relative efficiency of antioxidants can be expressed quantitatively [28] in terms of the ratio between the rates of the propagation reaction r_p_ (Equation (4)) and the rate of the inhibition reaction r_inh_ (Equation (6)), r_inh_/r_p_, so that if r_inh_/r_p_ > 1, then the antioxidant is efficient. Bravo-Díaz et al. [28] also showed that the inhibition reaction rate depends on the interfacial concentration of the antioxidants; thus, we determined them by employing Equation (15), seeking potential correlations between the effective concentrations of the antioxidants and their relative efficiency in halting the lipid oxidation reaction. The results in Figure 11 show that the variation in antioxidant efficiency and that obtained for the effective concentrations of AOs at the interface are parallel (finding a maximum for BG in both parameters) in the absence and the presence of HPCD. However, there is no correlation between both the effective AO concentrations in the aqueous and oil regions and the antiradical activity against the DPPH^•^ radical and their efficiency (Figure 11).

The results are in keeping with those previously reported for these and other antioxidants in different emulsions, and they stress the need to determine the effective concentration of AOs present at the interface to predict their efficiency in inhibiting lipid oxidation in an emulsified system.

Researchers have explored the use of phenolic esters such as alkyl gallates by forming cyclodextrin inclusion complexes of alkyl gallates [6,17,22]. These investigations mainly focused on the evaluation of the antioxidant and antibacterial properties of cyclodextrin –inclusion complexes of alkyl gallates. By controlling the acyl chain length of the gallate derivatives, the release behavior of the gallic acid and short-chain alkyl gallates from β-cyclodextrin was modulated in model everted rat gut sacs inclusion complexes [36].

Thus, it is well documented that cyclodextrins can host relatively hydrophobic molecules such as antioxidants in their cavities, forming inclusion complexes. We were, therefore, interested to discover the reasons why the addition of HPCD to our systems had no significant effect.

One potential hypothesis is that the observed negligible effect of HPCD is a consequence of the lack of formation of inclusion complexes because other chemicals may be interfering. In the present case, we hypothesize that the molecules of the surfactant Tween 20 may act as competitors for the HPCD cavity.

In attempting to shed some light on this point, we determined the inclusion constants of PG, BG, and OG with HPCD in the absence of Tween 20 and, for OG, in the presence of Tween 20. Those of PG and BG were evaluated by employing a solubility method, as described in Section 2.4.1; meanwhile, that of OG was determined by fluorescence because it is not water soluble.

**Figure 12:**
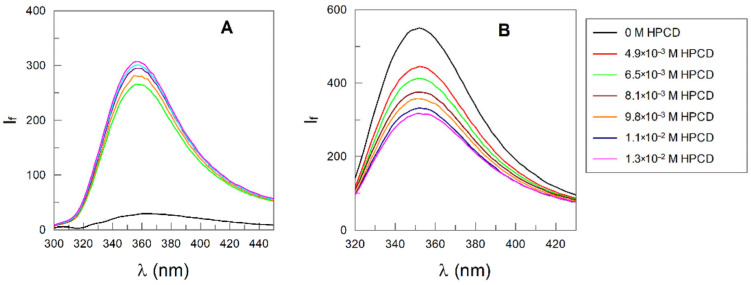
Fluorescence emission spectra of octyl gallate in the absence and presence of different concentrations of HP-CD (0.005–0.013 M) in citric–citrate buffer solutions of pH 3.5: (**A**) 0 M Tween 20 (λ_Exc_ = 296 nm, Ex.slit = 20 nm, Em slit 5 nm); (**B**) 0.0039 M Tween 20 (λ_Exc_ = 276 nm, Ex.slit = 5 nm, Em slit 5 nm).

Figure 12 shows selected emission spectra at different concentrations of HPCD in the absence and the presence of Tween 20.

**Figure 13:**
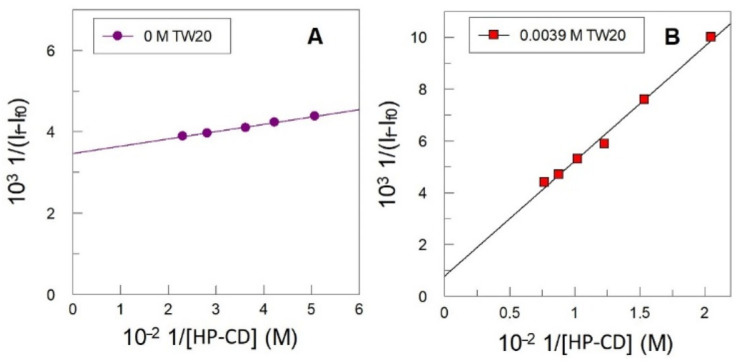
Double reciprocal curve between 1((I_f_–I_f0_) and 1/[HP-CD] for OG 7·10^−5^ M in citric buffer solution (0.04 M, pH 3.5) in the absence of Tween 20 (**A**) and in the presence of Tween 20 0.0039 M (**B**). Experimental conditions: T = 25 °C, (**A**) λ_Exc_ = 296 nm, Ex.slit = 20 nm, Em slit 5 nm; (**B**) λ_Exc_ = 276 nm, Ex.slit = 5 nm, Em slit 5 nm.

In the absence of Tween 20, an increase in [HPCD] increases the intensity of OG (Figure 12A); meanwhile, in the presence of Tween 20, an increase in [HPCD] leads to a decrease in the fluorescence intensity. Figure 13 shows the variation in the fluorescence intensity with CD, as well as the linear double-reciprocal plots of de 1/(I_f_-I_f0_) vs. 1/[CD]_Adeed_ (*n* = 1), from which the inclusion constant values displayed in Table 3 were obtained. *K*_C_ values increase upon increasing the chain length of the antioxidant, in keeping with the hydrophobic effect.

**Figure 14:**
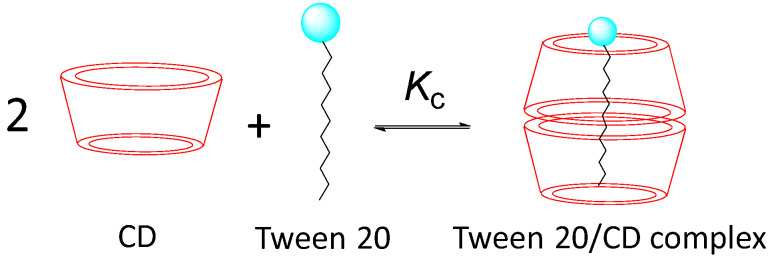
Representative formation of the inclusion complex Tween 20/CD (by assuming stoichiometry Tween–CD: 1:2), where *K*_C_ stands for the inclusion constant.

The much smaller value of OG in the presence of Tween 20 compared to that in its absence confirms that the main reason of the lack of HPCD effects is the blocking effect of the surfactant molecules, as illustrated in Figure 14.

It has long been understood that the addition of cyclodextrins (CDs) disfavors the self-assembly of surfactants in dilute solutions since the hydrophobic effect is destroyed upon the formation of the hydrophilic CD–surfactant inclusion complex [37,38].

Jiang et al. [39] reported that ionic surfactant–cyclodextrin inclusion complexes can spontaneously self-assemble depending on the total mass concentration. These self-assemblies were formed solely through H-bonds, although ionic repulsion is regarded as essential for maintaining their stability. Zhou et al. [40] found that self-assembly can occur for the 1:2 Tween 20:CD inclusion system even at concentrations of Tween 20 as low as 0.03 mM, indicating that the surfactant–CD inclusion complex may act as a building block for molecular self-assembly even in dilute solutions. Similar complexes were also found when different surfactants (Tween 40, 60, and 80 and Triton X100) were employed [41,42,43]. The results are therefore consistent with the literature reports indicating that Tween 20 forms 1:2 complexes with β-CD into CD, even at low Tween 20 concentrations, as illustrated in Figure 14.

## 4. Conclusions

We conducted a comprehensive study to quantify the effective concentrations of various gallate derivatives in the interfacial region of intact soybean oil-in-water emulsions. These emulsions were stabilized by the non-ionic surfactant Tween 20 and were prepared in both the absence and the presence of hydroxypropyl-β-cyclodextrin (HPCD). Our experimental approach allowed us to determine how much of each gallate was partitioned at the oil–water interface, which is a critical region for antioxidant activity in emulsified systems. The results showed a clear correlation between the antioxidant efficiency of each gallate derivative and its concentration at the interface: the antioxidant exhibiting the greatest interfacial concentration—specifically, butyl gallate—also provided the highest antioxidant efficiency. This finding highlights the importance of knowing the interfacial concentration of the antioxidants in maximizing their antioxidant effects in emulsified systems.

In addition to determining interfacial concentrations, we also examined how the presence of HPCD influenced the antioxidant distribution and its efficiency within these emulsions. Our data indicated that the presence of HPCD did not lead to any significant changes in the antiradical activity in bulk solution, in the distribution, and in the efficiency of the gallates within the emulsions. This lack of effect in the emulsions was observed consistently across all of the tested gallate derivatives, suggesting that the interaction between Tween 20 and HPCD at the oil–water interface may inhibit any potential enhancement in antioxidant performance that might otherwise result from cyclodextrin inclusion. In other words, the surfactant may compete with or block the cyclodextrin from effectively modulating the behavior of the antioxidants at the interface.

It is important to note that there is ongoing scientific debate regarding the formation of an antioxidant–cyclodextrin inclusion complex to enhance antioxidant efficiency. While some studies have reported positive effects, there is mounting evidence that the impact of cyclodextrins is highly dependent on the specific chemical structure of the antioxidant, the characteristics of the inclusion complex formed, and the environmental conditions within the system. Our findings contribute to this body of evidence by illustrating that cyclodextrin inclusion does not automatically translate to an improved antioxidant performance in all cases.

Ultimately, these results highlight the necessity of evaluating each emulsion system on a case-by-case basis. The benefits of cyclodextrin inclusion for enhancing antioxidant activity cannot be assumed to be universal; rather, it must be carefully assessed in the particular context of the antioxidants, surfactants, and environmental parameters involved. This understanding is essential for the rational design of more effective antioxidant delivery systems in food, pharmaceutical, and cosmetic emulsions.

## Figures and Tables

**Table 1:** Radical scavenging activity of gallates in the absence and presence of HPCD (1.1·10^−2^ M); T = 25 ± 1 °C (reaction time = 3600 s; aqueous citric acid/citrate (0.04 M, pH 3)–methanol 50:50, *v*/*v*).

	10^6^ × EC_50_ (M)	
	0 M HPCD	1.1·10^−2^ M HPCD
GA	8.36 ± 0.28	7.84 ± 0.08
PG	6.09 ± 0.12	5.57 ± 0.01
BG	6.02 ± 0.03	5.35 ± 0.04
OG	9.02 ± 1.05	8.27 ± 0.20

**Table 2:** Partition constants values in soybean oil–water binary mixtures (*P*_W_^O^) and in soybean oil-in-water emulsions (*P*_W_^I^ and *P*_O_^I^) for gallates in the absence and the presence of HP-CD; T = 25.0 °C.

		[HPCD] = 0		[HPCD] = 0.013 M	
AO	*P*_W_^O^	*P*_W_^I^	*P*_O_^I^	*P*_W_^I^ (app)	*P*_O_^I^
PG	0.85 ± 0.01	382 ± 18	450 ± 26	315 ± 14	371 ± 15
BG	3.25 ± 0.03	772 ± 33	238 ± 8	565 ± 9	174 ± 9
OG	---	---	26 ± 7	---	25 ± 5
LG	----	---	27 ± 9	---	25 ± 2

**Table 3:** Inclusion constants for the formation of the complex between gallates and HPCD in the absence and the presence of Tween 20.

Antioxidant	[Tween 20] (M)	*K*c (M^−1^)
PG	---	383 ± 12
BG	---	783 ± 35
OG	---	1946 ±85
OG	3.9 × 10^−3^	20 ± 5

## Data Availability

Data is contained within the article.
